# AltrexChimie, a Web Application for the Management and the Interpretation of Occupational Exposure Measurements to Chemical Substances

**DOI:** 10.3390/ijerph17103375

**Published:** 2020-05-12

**Authors:** Frédéric Clerc, Andrea Emili, Gautier Mater

**Affiliations:** Pollutant Metrology department, French National Research and Safety Institute for the Prevention of Occupational Accidents and Diseases (INRS), 1 rue du Morvan, 54500 Vandoeuvre-lès-Nancy, France; andrea.emili@inrs.fr (A.E.); gautier.mater@inrs.fr (G.M.)

**Keywords:** exposure assessment, measurement data, chemical risk, SEG

## Abstract

In most occupational settings, several chemical agents are commonly found, and the associated exposure risk for workers must be assessed. For this purpose, air samples can be collected and analyzed. AltrexChimie is a web application that helps industrial hygienists in the organization of the air sampling strategy and in the subsequent phases of data management, analysis, and communication. AltrexChimie contains a database of more than 550 chemical substances and their respective French Occupational Exposure Limit Values (OELV): Custom OELVs can also be defined by the user. AltrexChimie helps with the definition of key features of the sampling strategy, in particular by promoting a methodology for the design of Similar Exposure Groups (SEGs). Once measurement data are entered, they can be analyzed to obtain exposure diagnostics. Data management features allow for the easy storage and retrieval of measurements, and comprehensive dashboards help industrial hygienists (IHs) in the communication of results. Finally, with AltrexChimie it is also possible to assess exposure to multiple chemical substances and their additive effects. While most free software applications for the assessment of chemical exposure focus on the statistical computation of specific indicators, AltrexChimie offers several tools to assist IHs in the exposure assessment workflow.

## 1. Introduction 

In most occupational settings, several chemical agents are commonly found and the associated exposure risk for workers must be assessed. Exposure assessment is a multi-step procedure which consists in making an inventory of the chemical products used at the workplace, of their hazards and their use conditions followed by a chemical risk assessment to prioritize those work situations where risk is higher. In Europe, these rules aiming at assessing and managing risks are laid down in several European Directives [1,2,3]. The outcome of this assessment may be a “high”, “moderate”, or “uncertain” risk of exposure. If exposure risk is high, corrective actions must be undertaken in order to minimize it; if risk is moderate then the assessment needs to be regularly updated to ensure it does not increase with time; if risk is uncertain, the assessment needs to be refined. To assist in risk assessment, air samples are usually collected and analyzed.

The chemical substances targeted by air measurements are related either to possible long-term health effects, like carcinogenic substances with a dose–effect relationship, or to short-term effects, like irritant or neurotoxic substances. Conversely, poorly volatile substances with acute toxicity, sensitizers, or carcinogens without a dose–effect relationship are less likely to be targeted by measurements.

A sampling strategy defines the organization required to perform representative measurements of exposure by inhalation to chemical agents in the workplace. The concept was first introduced by US National Institute for Occupational Safety and Health in the 1970s [4] and subsequently adopted worldwide. In the EU, the EN 689 standard (updated in 2018 [5]) specifies general guidelines for establishing a sampling strategy, including the constitution of similar exposure groups (SEGs).

SEGs are the basic unit for establishing a sampling strategy. A SEG is a group of workers sharing a similar profile of exposure, meaning an exposure to the same chemicals, with the same intensity and variability. Industrial hygienists (IHs) use their expertise and their own perception of the work performed in the enterprise to define the SEGs. For each SEG in the enterprise, measurements are collected during a certain period of time to take into account possible variations related to operational conditions, as well as to other factors such as temperature or humidity.

Often, the aim of exposure measurements is to assess compliance with occupational exposure limit values (OELVs). Statistical inference is used to compute indicators allowing IHs to decide whether a work situation is compliant or not. In general, exposure to agents with chronic health effects is considered acceptable when less than 5% of all exposure measurements are above the OELV [5,6,7,8]. One of the mandatory conditions to ensure the quality of the diagnostic is the adequacy between the actual duration of the task performed, the duration of the sampling, and the type of OELV (acute or chronic toxicity) used for the comparison.

The computation of statistical indicators of exposure is made easier by the availability of several software tools. SAND and STAND are two software libraries for the R programming language [9] which include useful functions for the analysis of occupational data with non-detects. BWStats V3 is a web application that meets the requirements of the EN 689:2018 standard; it also includes many useful graphs and its older Microsoft Excel version V2 can still be downloaded for offline use [10]. Expostats is another web-based tool that implements Bayesian inference and proposes some innovative data visualizations [11]. IHStats is an Excel spreadsheet that uses a frequentist approach for compliance with the computation of the 95th percentile of the measurements’ distribution [12]. Hyginist is a downloadable Microsoft Windows executable file that implements a wide range of different statistical indicators to assess OELV compliance [13]. ProUCL is a Windows executable originally designed for environmental settings but fully useable for occupational health assessments [14]. IHDA is a Windows executable whose specificity is to allow IHs to include their own expert judgement in the assessment through Bayesian methods [15].

Finally, AltrexChimie (https://altrex.inrs.fr) was initially developed as a standalone program in 1993 and recently received a major update and migration to a web application. It computes statistical indicators and it manages exposure measurements at enterprise level. AltrexChimie is also linked to other tools for prior risk assessment such as Seirich [16]. AltrexChimie was developed at the French Research and Safety Institute for the Prevention of Occupational Accidents and Diseases (INRS), and is available in French and English. The aim of this paper is to show how the new version of AltrexChimie differs from the other tools available to IHs and how it can help them establish an efficient exposure assessment workflow.

The first part of the next section focuses on the data management functionalities of AltrexChimie, which represent the core innovation of the new version. The second part focuses on the definition of the sampling strategy and, in particular, on the procedure for SEG design. The third part describes the methodology for the assessment of multiple exposures to chemical substances and the statistical computations needed for establishing an exposure diagnostic for assessment of compliance with an OELV. In the results section, examples of the assessment procedure are given using AltrexChimie. The discussion highlights the tool’s limitations and suggests its possible use for biomonitoring data.

## 2. Materials and Methods 

### 2.1. The Management of SEGs and Measurements at the Establishment Level

For assessing occupational exposure, several SEGs are usually defined per establishment. For each SEG, several substances can be measured, leading to numerous exposure diagnostics. The definition of SEGs, the analysis of results and the diversity of diagnostics produced over years, and often by different people or contractors, represent a lot of information which is difficult to organize, store and retrieve. At the same time, IHs need to communicate efficiently to decision-makers at the enterprise level: Details on the statistical computation of the diagnostic are not of interest, results are.

**Figure 1:**
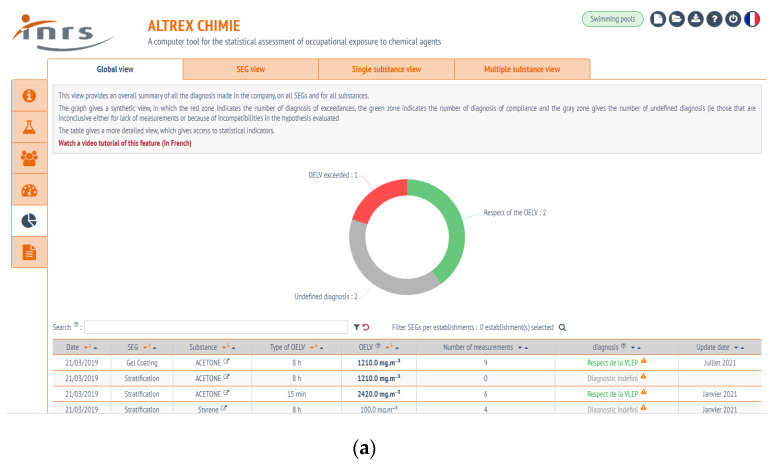

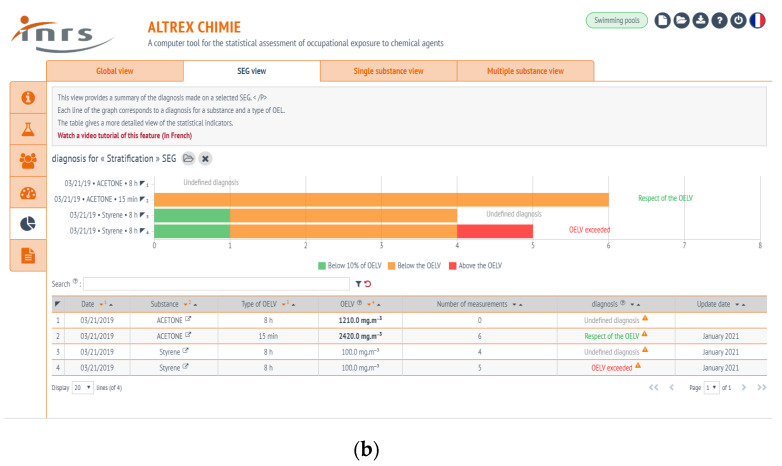
The dashboard shown in the upper part of the figure (**a**) presents a synthesis of all available diagnostics for the enterprise. In this example, one diagnostic exceeds the OELV, two are in compliance, and no conclusion can be made for the others. On the lower part of the figure (**b**), a synthesis of all diagnostics made for a similar exposure group (SEG) is indicated.

In AltrexChimie, the management of measurements is facilitated by an underlying database structure, linking the SEGs to substances, measurements and the corresponding diagnostics. This structure permits the creation of effective summary tables and dashboards, including compliant and non-compliant diagnostics, exposure diagnostics per SEG (including multiple exposure), and the list of substances correctly or poorly controlled. An overview of the computed diagnostics is presented in Figure 1 showing the “Dashboards” tab. Figure 1a is an overall view of all diagnostics, the green semicircles corresponding to compliant diagnostics, the red one to non-compliant diagnostics, and the grey one to other cases (in particular, situations where an insufficient number of measurements was available to define a diagnostic). The supporting data is shown in the table below the graph. Figure 1b shows the details of all available diagnostics for a specified SEG. Each horizontal bar in the graph represents a diagnostics made for the SEG: The bar length is proportional to the number of results and its color depends on the results compliance diagnostic (green for <10% of the OELV, orange for a value between 10% and the OELV, and red if >OELV).

Two additional views are available in the “Dashboards” tab: The first one introduces the substances and the SEGs and the second one presents the multiple exposure diagnostics.

These management functionalities are a core characteristic of AltrexChimie and they are not found in similar free software applications, which are primarily designed for the computation of statistical indicators from a data series.

### 2.2. SEG Design

An exhaustive exposure assessment of the entire worker population would require measurements carried out each day, on all workers, during all working shifts. Such a sampling strategy is clearly impossible to put into place. Therefore, the population’s features are inferred from the characteristics of the SEGs: Measurements from a SEG should be representative of both exposure levels and their random variability, which is made up of both a spatial and a temporal component.

Spatial variability is taken into account by using a map of the company, that is by identifying those areas within the enterprise where chemical substances are used. The map should be precise enough to identify work units, workplaces, and tasks performed at the workplace. Additional information related to the spatial variability of exposures can also be obtained, for example the differences in gestures and postures among workers.

The following five-step procedure is recommended for designing a SEG that considers spatial variability:

1. Define a hierarchical map of the enterprise that describes the different work units. The workstations are included in the work units. Each workstation has its own type of general ventilation and its own set of performed tasks. Each task has its type of emitting process and its own local exhaust ventilation (LEV);
2. List the chemical substances to be measured for each task;
3. List the jobs in the enterprise;
4. Define the SEG as different associations of work units/tasks/jobs;
5. Complete the definition of the SEG with additional specific information.

The temporal component of the variability accounts for the variation of exposure due to environmental conditions: e.g., high temperatures can increase the volatilization of substances, or workers may open doors and windows, increasing natural ventilation. Temporal variability also includes the random variations observed from one day to another. Therefore, measurements for each SEG have to be spread over a year, for all possible work shifts.

**Figure 2:**
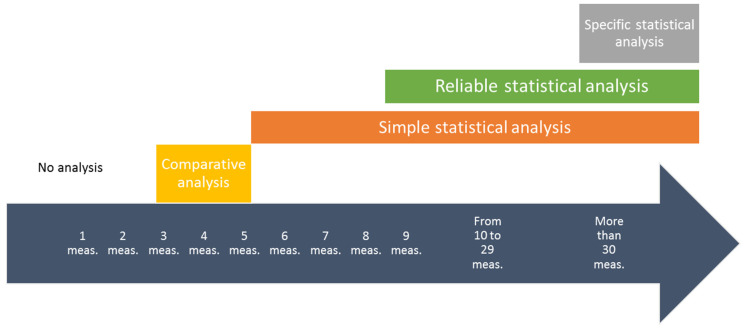
The number of measurements per SEG and the quality of the statistical analysis.

The number of measurements to be performed for each SEG is directly related to occupational health regulations, to standardization requirements, and to the internal procedures of the enterprise. On the other hand, the type of statistical analysis that can be applied to data is dependent on the number of measurements performed (Figure 2). Thus, the quality and the reliability of the diagnostic increases with the number of measurements.

**Figure 3:**
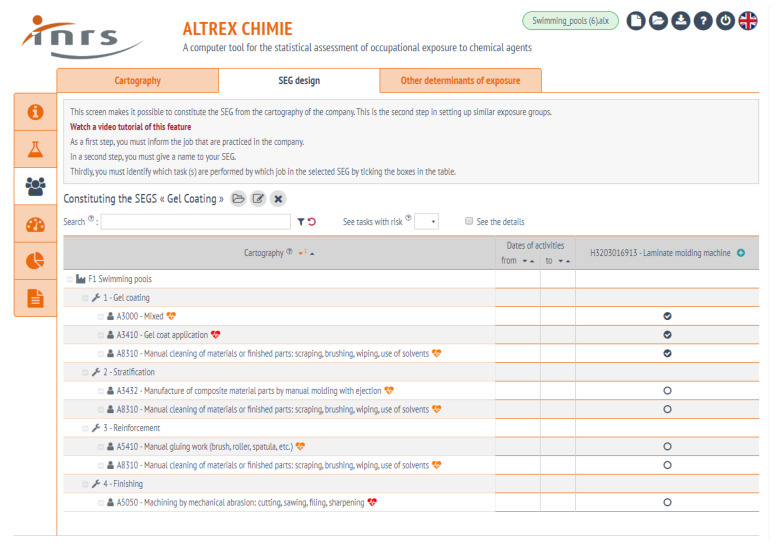
The design of SEGs: Hierarchical map of the factory listing the different fabrication steps; each task is associated with the substances and the definition of jobs. The last column shows where the tasks cross with jobs to define the SEG.

In AltrexChimie, the necessary steps for SEG design are implemented in three tab screens: “Map”, “SEG”, and “Determinants of exposure”. The “Map” tab implements steps 1 and 2 of the methodology (work units, workplaces, tasks, determinants of exposure and substances). The “SEG” tab implements steps 3 and 4: jobs and linking of the map with the jobs (Figure 3). The creation of supplementary determinants of exposure to the SEGs is implemented in the “Determinants of exposure” tab screen.

In other free software applications, the definition of the SEG is omitted most of the time, or it has to be a description not directly connected to the data series (commentaries, for example). In some tools, like Expostats or BWStats, it is possible to include determinants of exposure in the analysis and also to identify the workers linked with each measurement of the data series.

### 2.3. Mono and Multiple Exposure Assessment

The exposure assessment diagnostic in AltrexChimie relies on the assumption that results follow a lognormal distribution [5,17,18], that is, the logarithm of the values is normally distributed. Such distribution results from the multiplicative product of many independent positive random variables and it characterizes many observed natural and non-natural phenomena. For the correct treatment of data, the assumption of lognormality of exposure measurements must be verified by applying statistical tests such as the Shapiro–Wilk test of normality on the log-transformed variable.

Some additional hypotheses must be verified on the data series. The variability of measurements must be low enough to ensure that the SEG is indeed homogeneous. The Geometric Standard Deviation (GSD) is the preferred statistical indicator for this control: As a rule of thumb, a GSD lower than 3 is generally considered a good indicator of the SEG homogeneity [7]. The existence of sub-groups inside the SEG can also be verified by specific analyses, for example by running post-hoc tests in the context of an analysis of variance (ANOVA). Finally, the presence of temporal trends in data must be investigated: If exposure increases while the process is unchanged, the cause has to be found.

Among data, some values can be lower than the analytical limit of detection (LOD) or the limit of quantification (LOQ). These so-called left-censored data require specific statistical analysis which depends on their proportion [19,20,21]. When the data series is lognormally distributed, the preferred method for treating left-censored values is the Maximum Likelihood Estimator (MLE) [22,23]. The analytical uncertainty on measurements can generally be ignored because its impact on the exposure diagnostic is very low compared to the environmental variability of measurements [24,25].

Under a lognormal model, computed statistical indicators provide insights on the probability of measurements being in exceedance of the OELV. For example, if the 95th percentile of the probability distribution of concentration values is inferior to the OELV, then the expected proportion of measurements in exceedance of the OELV is lower than 5% [26]. The exceedance fraction is used as the preferred indicator of compliance.

In France, because of uncertainties inherent to the lognormal model, a 70% confidence interval on the exceedance fraction is also computed to assess compliance with regulatory OELVs [5,8,27]. Therefore, the comparison with the 5% threshold is made with the upper confidence limit (UCL) of this interval instead of the exceedance fraction itself. This procedure results in a more conservative approach to worker protection as it tends to overestimate exposure compared to the point estimate of the exceedance fraction.

The complete online report provided by AltrexChimie contains some additional graphs: The probability density, the log-log graph, and the box and whiskers plot. Data provided in the report include: The number of measurements and their type (short-term/long-term), descriptive statistics (arithmetic and geometric mean and standard deviation, range, …), the Shapiro–Wilk statistic and its p-value (to assess the log-normality), Spearman’s test and its p-value (to assess the temporal drift), an ANOVA for each determinant of exposure and the exposure diagnostic (exceedance fraction and its 70% confidence interval).

When multiple substances having the same toxic effects are measured for a SEG on the same day, a sum of the measurement/OELV ratios calculated for each substance can be computed. The result of this computation is a multiple exposure index, which is considered indicative of the possible combined toxic effects of the mixture of substances to which workers could be exposed in the described situation, assuming additive effects [28]. In practice, an index greater than 1 indicates a risk of multiple exposure. This kind of assessment requires additional toxicological information on the substances, and the analysis is carried out with a purpose-designed tool such as MiXie [29,30].

If the result of the exposure diagnostic is non-compliance with the OELV, then corrective actions must be performed in order to suppress or reduce exposure. If the result is compliance with the OELV, then periodical controls must be regularly performed, in order to ensure that the process is well controlled. The lognormal model can be used to provide indications on the optimal frequency of these controls. The higher the exceedance fraction, the more frequent the controls. In AltrexChimie, the value of the multiple exposure index is used like an exposure measurement value, thus allowing its statistical treatment.

No other free software provides explicit multiple exposure assessment functionalities. Meanwhile, all of them can be used with multiple exposure indexes instead of measurements. The statistical computations offered by AltrexChimie are among the simpler ones, like the ones proposed by BWStat, IHStats, or the R libraries SAND and STAND. Hyginist offers a greater number of statistical indicators, allowing for a comprehensive assessment for experts. Expostats and IHDA offer Bayesian-based computations, which facilitate the communication of results under a probabilistic perspective; IHDA also allows prior expert judgment to be included in the computation.

## 3. Results

### Examples of Application Using AltrexChimie

To better illustrate the different functionalities offered by AltrexChimie, two examples of application are proposed: They concern (1) the manufacture of swimming pools and (2) rotogravure.

The manufacture of swimming pools using composite materials is a major activity in France: The related industrial sector, “Plastic products manufacture for construction” includes more than 1000 enterprises [31]. Manufacture starts by coating the plastic mold with a special gel; layers of fiberglass mats are then applied on the coated mold, and fixed with resin; reinforcement plastic parts are subsequently glued to the structure and, finally, the pool is finished by sanding and grinding so as to smooth surfaces and remove rough edges. This industrial process presents a high chemical risk due, in particular, to the use of styrene, acetone, and the emission of inhalable dust.

**Figure 4:**
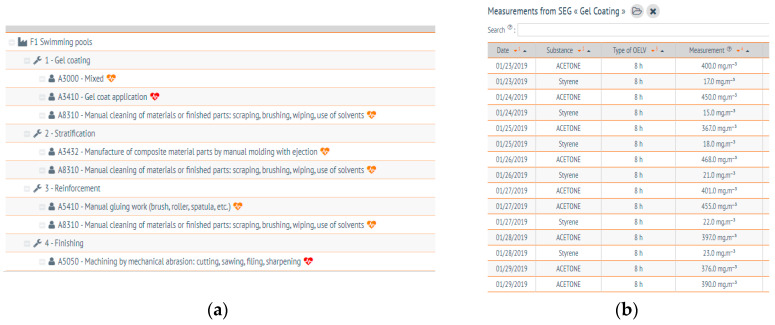

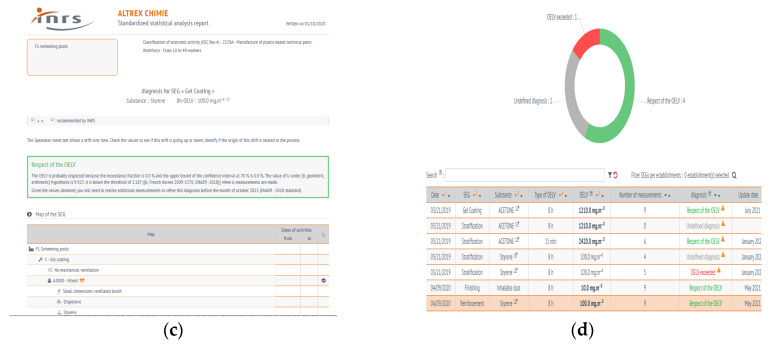
Assessing exposure during the making of swimming pools with AltrexChimie. On the top (**a**), the cartography and the task based SEGs. On the top (**b**), example of measurement data series related to gel coating SEG. On the bottom (**c**), an example of the report containing the exposure diagnostic and the statistical indicators of styrene measurements for the same SEG. On the bottom (**d**), the produced dashboards for all four SEGs and the different substances.

In the following example, four task-based SEGs are designed, considering the manufacturing process and the map of the establishment: Gel coating, stratification, reinforcement, and finishing (Figure 4a). For each SEG, the results of exposure measurements are added (Figure 4b). Measurements can concern any type of substance defined in AltrexChimie, and any type of OELV (short-term or 8 h) but the user must verify that the measurement sampling time is in accordance with the OELV. An exposure diagnostic can be computed for each series of measurements performed for the different substances in the SEG and their respective OELVs. Results are given by AltrexChimie in the form of a printable report, complete with graphs, with examples provided in Figure 4c. Finally, the diagnostics at the establishment level are shown in Figure 4d.

**Figure 5:**
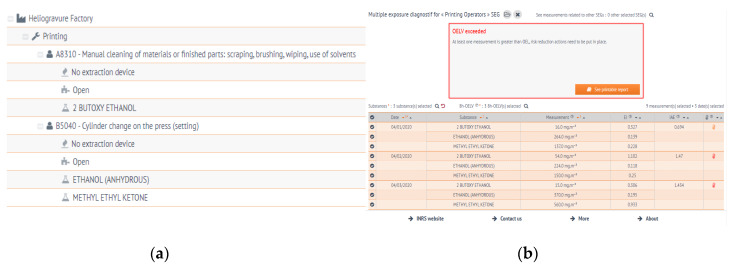

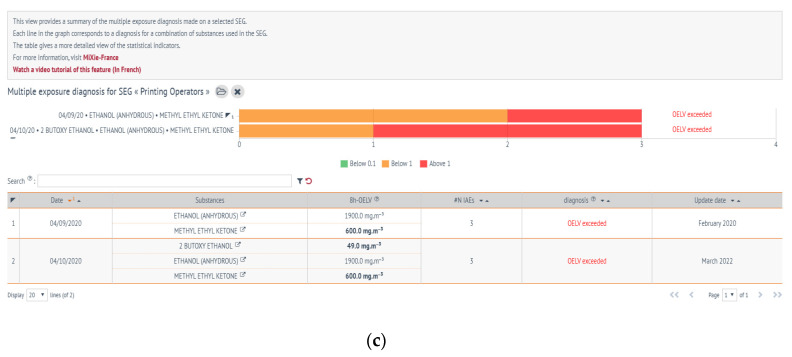
Assessing exposure during maintenance tasks in the printing industry with AltrexChimie. On the top (**a**), the cartography and the SEG, with the details of the protective equipment and substances shown. On the top (**b**), example of multiple exposure diagnostic with three substances. On the bottom (**c**), an example of the multiple exposure dashboard with two exposure diagnostics, for different combinations of substances.

The printing industry represents around 3000 companies and employs almost 40,000 workers in France. The inks in use are composed of mixtures of pigments and solvents. The printing machines are composed of engraved rotating cylinders, which apply the ink on a large coil of paper at high speed. Once the ink has dried, the coil is cut and folded to produce different types of publications, including newspapers, booklets, or catalogues. When the production changes, the printing cylinders are replaced in the machine and cleaning and maintenance operations are performed, during specific work shifts. During these operations, workers are exposed to some solvents. In this example, the solvents are ethanol, methyl ethyl ketone (MEK) and 2-butoxyethanol. Only one SEG is defined (“Printing Operators”) and its collective protection equipment are shown on Figure 5a. Three measurements for each solvent were performed and a multiple exposure diagnostic was computed, showing that the workers are probably overexposed (Figure 5b).

With AltrexChimie, it is possible to compute several multiple exposure diagnostics for a SEG, depending on the different combinations of substances. In Figure 5c, a multiple exposure diagnostic composed of 2 substances (ethanol and methyl ethyl ketone) is shown in the first line of the histogram, while another one composed of 3 substances (2-butoxyethanol, ethanol, and methyl ethyl ketone) is shown in the second line.

When using AltrexChimie to compute multiple exposure diagnostics, the appropriate combination of substances has to be carefully defined: The toxicological effects of the substances must be considered additive on selected target organs. Depending on their toxicological properties, all the measured substances may not be assessed at the same time. Groups may be formed by toxicological classes and a substance may contribute to several classes. For more information, refer to MiXie [29,30]. 

## 4. Discussion

To support IHs, AltrexChimie provides a list of 567 substances, complete with OELVs, EU’s regulatory hazard statements (under the “Classification, Labelling and Packaging—CLP Directive”) and INRS’s toxicological and technical fact sheets. Users can also import or create their custom list of substances, complete with alternative limit values for non-regulatory assessments.

The regulatory context in France does not take into account important parts of exposure assessment. In particular, assessment relies on the exceedance fraction computed on the lognormal hypothesis, but the regulation does not make it mandatory to verify this hypothesis. AltrexChimie compensates this gap by automatizing this verification. However, as a rule, results of the exposure assessment should always be subject to critical evaluation and the (in)adequacy of the diagnostic with prior risk assessment should be verified and analyzed.

The requirements of the concept of threshold versus the concept of an estimate interval, such as the result of a Bayesian analysis [11,32,33] should be questioned. Such estimate intervals are generally less precise but more realistic. In this case, the expert judgement of the industrial hygienist becomes dramatically important. However, in a highly regulated context such as in France, this alternative may not be currently applicable. In AltrexChimie, measurements and statistical computations comply with the European EN 689 standard. The standard recommends the collection of at least six measurements per SEG to compute a reliable exposure compliance diagnostic. Meanwhile, a simpler procedure is also proposed when only three to five measurements are available. This procedure is also implemented in tools such as BWStats and, partially, in Hyginist and IHStats. Conversely, the standard does not provide guidance when the number of available measurements is large, for example greater than 30. This situation occurs more frequently in large companies, because the regulation makes periodical controls mandatory. When a sufficient number of measurements is available, more specific and purpose-designed statistical procedures should be used.

Because of the variability in exposure levels in the workplace, the odds of observing a measurement with a concentration greater than the OEL increases with the number of measurements. Thus, when a high number of measurements is available, exposures above the OEL are generally observed. In this case, an enterprise complying to the EN 689 would be required to improve the process or the risk mitigation equipment, even if the exceedance fraction is low and its value reliable, because of the high number of measurements. This is an intrinsic contradiction of the proposed procedure that clearly highlights the lack of guidance when a substantial number of measurements is available. This explains the computational choices made in AltrexChimie, which are different to IHDA or Expostats.

One major issue during the development of the new version of AltrexChimie as a web application was the protection of user data. While an online tool is less limited by operating systems and updates, an offline tool is more reassuring to the user because important data are not transmitted over the network. To guarantee data confidentiality, innovative software functionalities were implemented, so that users can keep their data on their own computers, and no data is stored on the INRS servers. Other online tools such as Expostats and BWStats V3 do not provide a fully structured data storage system: Only screenshots and copy/pasting are allowed. 

The possibility of publishing an open-source software library derived from AltrexChimie was also discussed during the development phase. Some companies already have measurement management tools, either purpose-designed or included in laboratory information management software and they may therefore be interested in upgrading by adding only AltrexChimie’s statistical computation engine. This possibility was rapidly excluded, because such a library would not provide significant advantages over already existing packages such as STAND, which can be used instead of AltrexChimie in this particular context.

Finally, some important limitations of AltrexChimie are related to the choices made for the multiple exposure assessment. First of all, the toxicological information of the effects of substances on target organs is not included in AltrexChimie, and the effects are only considered additive. More importantly, the statistical procedure used to compute a multiple exposure diagnostic is the same as for a single substance. This hypothesis does not take into account important issues both in toxicological terms like the toxicokinetics of substances in the body and in statistical terms like the possibility of multimodal distributions of exposure measurements. This feature requires more research work to be fully reliable but it is a first step towards a statistical assessment of multiple exposure measurement data.

## 5. Conclusions

Measurement-based exposure assessment is the final step in chemical risk assessment, which is applicable when regulations make it mandatory (in the case of carcinogenic, mutagenic, and reprotoxic substances, for example) or when other simpler and cheaper methods fail to conclude on the risk posed by a work situation. Exposure assessments must be carefully planned, organized, and carried out in order to obtain reliable results to support conclusions on the risk of workers. Thus, the adopted assessment methodology is of paramount importance. Because this methodology relies on complex expert statements and hypotheses, it is important to have suitable tools to put it into practice.

In this paper, we presented the methodology implemented in AltrexChimie for the collection and interpretation of chemical substances measurement data, obtained from air sampling in occupational settings. AltrexChimie implements the statistical models required for the computation of an exposure diagnostic with regards to a limit value. The software provides assistance for the design of similar exposure groups (SEGs), a set of workers whose exposure to chemical substances is assumed to be similar. It also provides all the functionalities for the management of the SEGs, the measurements, and the diagnostics. Industrial hygienists must use complex methods to assess exposure, but they also have to communicate the results in a relevant manner. Indeed, they must convince decision-makers to engage in the required preventive actions. AltrexChimie offers comprehensive dashboards, which can be copy/pasted. Finally, AltrexChimie allows assessment of exposure related to a mixture of substances by hypothesizing that the effects on target organs are additive. AltrexChimie can be accessed from altrex.inrs.fr; it does not require users to log in, and user data is stored on the user’s computer only.

Several improvements of the exposure assessment methodology and the associated tools can be envisioned. Among these, a simple procedure for handling left-censored data (measurements below the LOD); guidelines for analyzing SEGs with large measurement datasets, using more specific models; recommendations on the use of the procedure for biometrology data. Finally, a shift towards the Bayesian methodology with its underlying assessment based on uncertainty would certainly allow both more realistic (yet less accurate) assessments and provide a greater use of the knowledge and expert judgment of industrial hygienists in the overall assessment, as opposed to simply applying statistical routines to analyze measurement data.
